# The influence of inorganic nitrogen fertilizer forms on micronutrient retranslocation and accumulation in grains of winter wheat

**DOI:** 10.3389/fpls.2013.00320

**Published:** 2013-08-16

**Authors:** Nunun Barunawati, Ricardo F. Hettwer Giehl, Bernhard Bauer, Nicolaus von Wirén

**Affiliations:** Molecular Plant Nutrition, Department of Physiology and Cell Biology, Leibniz Institute of Plant Genetics and Crop Plant ResearchGatersleben, Germany

**Keywords:** biofortification, phytosiderophores, ammonium, nitrate, iron, zinc

## Abstract

The fortification of cereal grains with metal micronutrients is a major target to combat human malnutrition of Fe and Zn. Based on recent studies showing that N fertilization can promote Fe and Zn accumulation in cereal grains, we investigated here the influence of nitrate- or ammonium-based N fertilization on the accumulation of Fe, Zn, and Cu as well as metal chelator pools in flag leaves and grains of winter wheat. Fertilization with either N form increased the concentrations of N and of the metal chelator nicotianamine (NA) in green leaves, while 2'-deoxymugineic acid (DMA) remained unaffected. Despite the differential response to N fertilization of NA and DMA levels in flag leaves, N fertilization remained without any significant effect on the net export of these metals during flag leaf senescence, which accounted for approximately one third of the total Fe, Zn, or Cu content in leaves. The significant increase in the accumulation of Fe, Zn, and Cu found in the grains of primarily ammonium-fertilized plants was unrelated to the extent of metal retranslocation from flag leaves. These results indicate that an increased N nutritional status of flag leaves promotes the accumulation of Fe, Zn, and Cu in flag leaves, which is accompanied by an increased pool of NA but not of DMA. With regard to the far higher concentrations of DMA relative to NA in leaves and leaf exudates, DMA may be more relevant for the mobilization and retranslocation of these metals in high-yielding wheat production.

## Introduction

The amount of nutrients that accumulates in plant fruits or seeds depends mainly on the availability of each particular element in the soil and on the plant's ability to acquire and translocate these nutrients to the respective sink organs. Unfortunately, not much of the plant-derived food contains sufficient amounts of nutrients to meet the dietary requirements of humans. In particular cereal grains are inherently poor in iron (Fe) and zinc (Zn), although they are excellent sources of calories (White and Broadley, 2005; Cakmak, 2008; Newell-McGloughlin, 2008). Thus, a diet which is mainly based on cereals can lead to micronutrient malnutrition and contributes to the fact that almost half of the world's population suffers from diseases related to the deficiencies of Fe and/or Zn (WHO, 2002; Nestel et al., 2006). In this context, the improvement of Fe and Zn contents in cereal grains has become a high-priority research area (White and Broadley, 2005; Bouis et al., 2009). Recently, it has been demonstrated that strategies for micronutrient biofortification in plants are becoming economically efficient and suitable to combat micronutrient malnutrition in developing countries (Stein et al., 2007).

A substantial part of the nutrients accumulating in the vegetative tissue of a cereal plant is remobilized into the developing grains (Wiedemuth et al., 2005), a process that is induced during senescence in source tissues, in particular in leaves and stems (Gregersen et al., 2008). Relative to macronutrients like N, P, and K, micronutrients are generally remobilized to a lower extent (Marschner, 2012). While only 20% of the ^59^Fe applied to bean leaves was exported to sink leaves, this amount increased to 34% when senescence was induced by shading of ^59^Fe-supplied leaves (Zhang et al., 1995). In addition, it has recently been shown that N deficiency- or shading-induced leaf senescence enhances Fe mobilization in old leaves and favors Fe retranslocation from source to sink leaves in vegetatively growing barley plants (Shi et al., 2012). Moreover, the proportion of retranslocated nutrients is highly dependent on the micronutrient's mobility in the phloem. For instance, Fe has a comparatively moderate mobility in the phloem (Kochian, 1991), whereas Zn is highly phloem-mobile (Marschner, 2012) and up to 70% of the Zn from the vegetative parts of wheat plants can be retranslocated into the grains (Grusak et al., 1999).

During the retranslocation from source to sink tissues, Fe and Zn are transported through the phloem most likely in complexed forms. It is assumed that complex formation avoids precipitation of Fe and Zn as a consequence of the slightly alkaline pH and the relatively high P concentrations found in the phloem sap (Briat et al., 2007; Curie et al., 2009). In many plant species, nicotianamine (NA) appears to be the major chelator for Fe during retranslocation via the phloem, because (i) NA is able to form stable complexes with both Fe(II) and Fe(III) at neutral pHs (von Wirén et al., 1999); (ii) a decrease of NA levels in NA aminotransferase-expressing tobacco plants impaired Fe loading into seeds (Takahashi et al., 2003); and (iii) an increase in NA synthesis by activation tagging of a NA synthase (NAS) gene in rice led to higher Zn concentrations in transgenic rice (Lee et al., 2011). In graminaceous plant species, besides NA also phytosiderophores may play an important role during micronutrient transport in the phloem (Inoue et al., 2008). This is based on the observations that the phytosiderophore 2'-deoxymugineic acid (DMA) has been detected in the phloem sap of barley plants (Mori et al., 1991) and that Fe-DMA and Zn-NA complexes were found in the phloem sap of rice (Nishiyama et al., 2012). Since chelation plays such an important role during the long-distance transport of metal micronutrients, strategies for improving micronutrient contents in grains should also consider how they affect the pool of micronutrient chelators. With regard to a role in metal retranslocation, citrate has so far received little attention and only been considered as relevant for root-to-shoot translocation of Fe in the xylem (Rellan-Alvarez et al., 2010), although recent experiments indicated a substantial increase in Fe-citrate complexes in senescing barley leaves (Shi et al., 2012). This observation pointed to a likely involvement of citrate in the mobilization of Fe as a prerequisite for subsequent retranslocation.

One option to enhance micronutrients in grains is to supply crop plants with micronutrients in highly soluble forms by soil or foliar application. However, under most circumstances this strategy is costly and its effectiveness is often restricted to specific soil conditions and plant cultures (White and Broadley, 2009). Alternatively, the manipulation of nitrogen (N) nutrition can have a significant effect on the retranslocation of Fe and Zn in cereals. Nitrogen nutrition is assumed to have a positive effect, because N is required for the biosynthesis of NA and the Fe transport peptide ITP (Krüger et al., 2002; Shi et al., 2012). Additional supply of N has been shown to enhance the accumulation of Fe and Zn in wheat grains (Kutman et al., 2010, 2011; Shi et al., 2010; Aciksoz et al., 2011; Erenoglu et al., 2011). In part, this beneficial effect is related to the fact that the supply of sufficient amounts of N increases the grain protein concentrations, thereby increasing the sink strength of grains for Fe and Zn. In line with this, previous reports have shown that protein concentrations correlate positively and significantly with Fe and Zn concentrations in grains (Peleg et al., 2008; Zhao et al., 2009). In addition, the N status can also affect the remobilization of micronutrients, since Fe export out of source leaves was inhibited under N-sufficient but stimulated under N-deficient growth conditions (Shi et al., 2012). Altogether these studies indicate that N management represents a promising agronomic strategy to improve micronutrient contents in wheat grains.

So far, most of the studies assessing the relation between N nutrition and micronutrient accumulation in grains were carried out in hydroponics or in the greenhouse (Aciksoz et al., 2011; Erenoglu et al., 2011; Kutman et al., 2011, 2012; Carlisle et al., 2012; Shi et al., 2012). To assess the practical relevance of N fertilization for improved grain metal concentrations, also field studies have been conducted which mostly support a positive effect of N on grain Fe or Zn accumulation in particular when root activity held on and Fe or Zn were supplied (Shi et al., 2010; Xue et al., 2012). However, most of the studies in wheat were conducted on sites conferring rather low or moderate grain yields (Shi et al., 2010; Xue et al., 2012), leaving it open whether also the important goal of Fe and Zn biofortification in cereals can also be attained under high-yielding conditions. Another aspect that has remained poorly investigated under field conditions is the impact of different inorganic N forms on micronutrient accumulation in grains. In most agricultural soils, nitrate (NO^−^_3_) and ammonium (NH^+^_4_) are the most abundant inorganic N forms. Besides serving as N source, these two N forms do also distinctly affect many physiological processes such as changes in rhizosphere pH or in the synthesis of organic acids which, in turn, may act as micronutrient chelators (Zou et al., 2001; Marschner, 2012). In a recent study, it has been reported that, compared to nitrate, the supply of ammonium to hydroponically-grown wheat plants resulted in a general increase in the concentrations of many nutrients, including Fe, Cu as well as Zn, and particularly bioavailable Zn (Carlisle et al., 2012). These results are likely related to the fact that nitrate and ammonium can cause distinct or even opposing effects on the pH of the rhizosphere and the apoplast of root or leaf cells as well as on the uptake, translocation and remobilization of micronutrients (Zou et al., 2001; Marschner, 2012). However, it is not yet fully understood whether nitrate or ammonium affects also other processes relevant for micronutrient accumulation in grains, such as flag leaf senescence or the synthesis and pool size of metal chelators.

Thus, the present study was designed to investigate the influence of a primarily nitrate- or ammonium-based N fertilization on the accumulation of micronutrients in the grains of field-grown winter wheat cultivated under high-yielding conditions. Particular attention was placed on the effect of these N forms at the onset of flag leaf senescence and its consequence for the availability of major metal ligands in flag leaves and leaf exudates.

## Materials and methods

### Plant material and growth conditions

**Figure 1:**
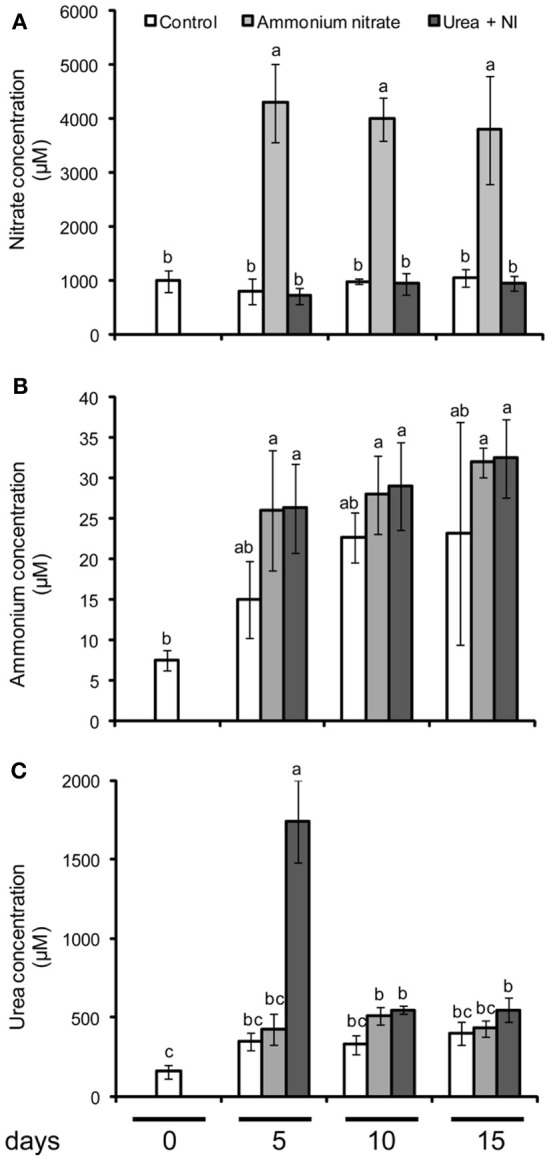
**Concentration of nitrate (A), ammonium (B), and urea (C) in soils after the fertilization of ammonium nitrate or urea + nitrification inhibitor (NI).** Control soils did not receive any N fertilization. Shown are means ± SD (*n* = 4) Different letters indicate significant differences according to Tukey's test (*P* ≤ 0.05).

Winter wheat (*Triticum aestivum* cv. Akteur) was grown on a gley tscherniza (Table 1) using common agriculture practices. Cation exchange capacity (CEC) was determined in 2 mm-sieved soil, extracted by 1 M ammonium chloride, pH 7.0, and considering the sum of protons, Ca, Mg, K, and Na. Soil N was determined in dry-ashed soil samples following the Dumas principle. Micronutrients were determined by ICP-OES using CaCl_2_/DTPA extracts. Soil analysis was performed by a certified lab (Agrolab, Oberdorla, Germany). The field experiment was arranged in 30 m^2^ plots in a randomized block design, in which each treatment was represented once in four blocks, from which 10 plants were sampled in the middle of each plot for analyses. Before sowing, all plots received 40 kg N ha^−1^ in the form of (NH_4_)_2_SO_4_. At anthesis (EC65), fertilizer treatments consisted of no additional supply of N (control) or a broadcast application of 80 kg N ha^−1^ in the form of either ammonium nitrate (nitrate-based treatment) or urea plus the nitrification inhibitor dicyandiamide (ammonium-based treatment). These fertilizer N forms were employed because undesired side effects of accompanying salts in the N fertilizer could be circumvented. N forms in the soil solution were monitored in soil samples collected 5, 10, and 15 days after fertilizer application and sieving through a 2 mm mesh. Then, 10 g of the soil sample were extracted by 40 mL of a 25 mM CaCl_2_ solution for 1 h and shaking at 220 rpm. After centrifugation (20 min, 4500 rpm, 4°C) the supernatant was analyzed for ammonium (Husted et al., 2000), nitrate, and urea (Kyllingsbaek, 1975) (Figure 1). To monitor changes in micronutrient contents in response to N fertilizer forms, flag leaves were harvested when they were still fully green (EC75) and, a second time, when they started to exhibit symptoms of senescence (EC85). Leaves from both developmental stages were used for chlorophyll, element and metabolite analysis or for the collection of leaf exudates. Spikes were harvested together with flag leaves at EC75 but for the second harvest at full maturity (EC99) to allow micronutrient re-allocation to be completed.

**Table 1:** **Selected soil characteristics and nutrient concentrations of the experimental field used in this study**.

**Type of soil**	**CEC (mmol kg^−1^)**	**Organic matter (%)**	**pH (CaCl_2_)**	**N (%)**	**P**	**K**	**Fe**	**Zn**	**Mn**	**Cu**
					**(μg g^−1^)**					
Silty to clayey loam	122	2.4	7.3	0.13	59	86	2.4	2.2	15	1.6

Soil samples were taken from a depth of 0 to 90 cm. Micronutrient concentrations were measured in CaCl_2_/DTPA extracts.

### Chlorophyll determination

Total chlorophyll levels were assessed in flag leaves by following the protocol described in Lichtenthaler (1987). Briefly, 80% ethanol was added to 20 mg homogenized leaf samples and incubated at 80°C for 60 min. Samples were allowed to cool down for 15 min and centrifuged at room temperature. Double-distilled water was added to the supernatant and homogenized. Chlorophyll concentrations were measured by spectrophotometry (Guebel Instrumentelle Analytik, UVikon XL Biotek Instrument, Germany) at 652 nm.

### Collection of leaf exudates

In order to collect the exudates of flag leaves, single flag leaves were cut at their base and incubated immediately in a buffering solution containing 15 mM EDTA at pH 7.5 as described in Weibull et al. (1990). Leaves were incubated in a growth cabinet (HPS 1500/S, Heraeus, Germany) for exudate collection over a period of 3 h at 21°C, 98% humidity and a light intensity of 120 μmoles m^−2^ s^−1^.

### Determination of nitrogen and micronutrients

Nitrogen concentrations were determined in dried and ground samples of flag leaves or grains using an elemental analyzer (EA3000; Hekatech Germany). The concentrations of the micronutrients Fe, Zn, and Cu in flag leaves and in grains were measured by inductively-coupled plasma optic emission spectrometry (ICP-OES; iCAP, Thermo Scientific, Germany) after wet digestion of sample powder with nitric acid in a high-pressure digestion apparatus (UltraClave III, MLS, Leutkirch, Germany). For quality control, a certified reference material from the National Institute of Standards and Technology (Gaithersburg, USA) was used.

### Sample fractionation and chelator analysis

The levels of NA, DMA, and citrate in flag leaves and flag leaf exudates were determined exactly as described in Shi et al. (2012). Deep-frozen flag leaves were homogenized and 50–100 mg was used for extraction without derivatization. Extraction was made by adding 0.5 ml 80% ethanol. Samples were then incubated at 80°C for 60 min and cooled down for about 15 min. After centrifugation, the supernatant was removed and evaporated under vacuum at 55°C. Samples were resuspended in 250 μ l ultrapure H_2_O. The separation of organic acids was carried out using an AS11-HC column (2 × 250 mm) connected to an AG11-HC (2 × 50 mm) column and an ATC anion trap column. The gradient was accomplished with ultrapure water and increasing concentrations of KOH from a concentrated EluGen Catride EGC-KOH (Dionex, Idstein, Germany) and Eluent Generator EG40. The column was equilibrated at a flow rate of 0.25 ml per minute with 4% KOH.

### Amino acid determination

For amino acid determinations, samples were prepared and concentrated under vacuum as described above. Then, 250 μl of ultrapure H_2_O was used to resuspend the samples. The ACQ reagent (aminoquinolyl-N-hydroxysuccimidyl carbamate) was used for derivatization at 55°C for both leaves and leaf exudates (Cohen and Michaud, 1993). Amino acids were determined by HPLC mounted to an ALT2, Waters 2795 separation module and a multi fluorescence detector (model 2475, Waters GmbH, Eschborn, Germany). The reagents reacted with primary and secondary amino acids to yield highly stable urea that fluoresce strongly at 400 nm after passing through a xBridg™ C18 3 μm column. The buffer contained 140 mM sodium acetate, adjusted by acetic acid (Suprapur Merck, Germany) to pH 5.8, and 7 mM triethanolamine (Sigma, Germany), acetonitrile (Roti C Solv HPLC, Roth) and ultrapure H_2_O. Data collection and calculations were carried out by the Empower software (Waters GmbH, Eschborn, Germany).

### Data analysis

Depending on the number of factors assessed, data were subjected to a One-Way or Two-Way ANOVA to assess the effect of treatments and their interactions on the analyzed traits (Table 2 and Table A1). Significant differences between means were then determined using the Tukey's test at 95% confidence (*P* ≤ 0.05).

**Table 2:** **One-Way or Two-Way analysis of variance (ANOVA) of the effects of N fertilization, leaf developmental stage and their interactions on response variables of field-grown winter wheat (*Triticum aestivum* cv. Akteur)**.

	**N fertilization**	**Leaf dev. stage^a^**	**N fertil. × leaf dev. stage**
Dry weight of flag leaves	0.358	0.058	0.283
Chlorophyll concentration	0.015	<0.001	0.974
N content of flag leaves	<0.001	<0.001	0.473
Thousand grain weight^b^	–	0.385	–
Grain yield^b^	–	0.350	–
N content of grains	0.001	<0.001	0.004
Fe content of flag leaves	0.487	<0.001	0.143
Zn content of flag leaves	0.505	<0.001	0.633
Cu content of flag leaves	0.089	<0.001	0.483
Fe content of grains	<0.001	<0.001	<0.001
Zn content of grains	0.038	<0.001	0.019
Cu content of grains	0.045	<0.001	<0.001
NA concentration in flag leaves	<0.001	<0.001	0.003
DMA concentration in flag leaves	0.521	0.209	0.697
Citrate concentration in flag leaves	0.024	<0.001	<0.001
NA exudation rate from flag leaves	0.206	0.002	0.017
DMA exudation rate from leaves	0.090	<0.001	0.001
Total free amino acids in flag leaves	0.037	<0.001	0.303
Glutamine concentration in flag leaves	0.010	<0.001	<0.001
Asparagine concentration in flag leaves	<0.001	<0.001	<0.001
Glutamate concentration in flag leaves	0.184	<0.001	0.158
Aspartate concentration in flag leaves	0.007	<0.001	0.081

The F-value probabilities at 95% confidence are indicated.

a Refers to the developmental stage of either flag leaves (green or senescent) or grains (immature or mature).

b Assessed only at one harvest point (mature grains). Data was subjected to One-Way ANOVA.

## Results

### Influence of N fertilization on N forms in the soil solution

**Figure 2:**
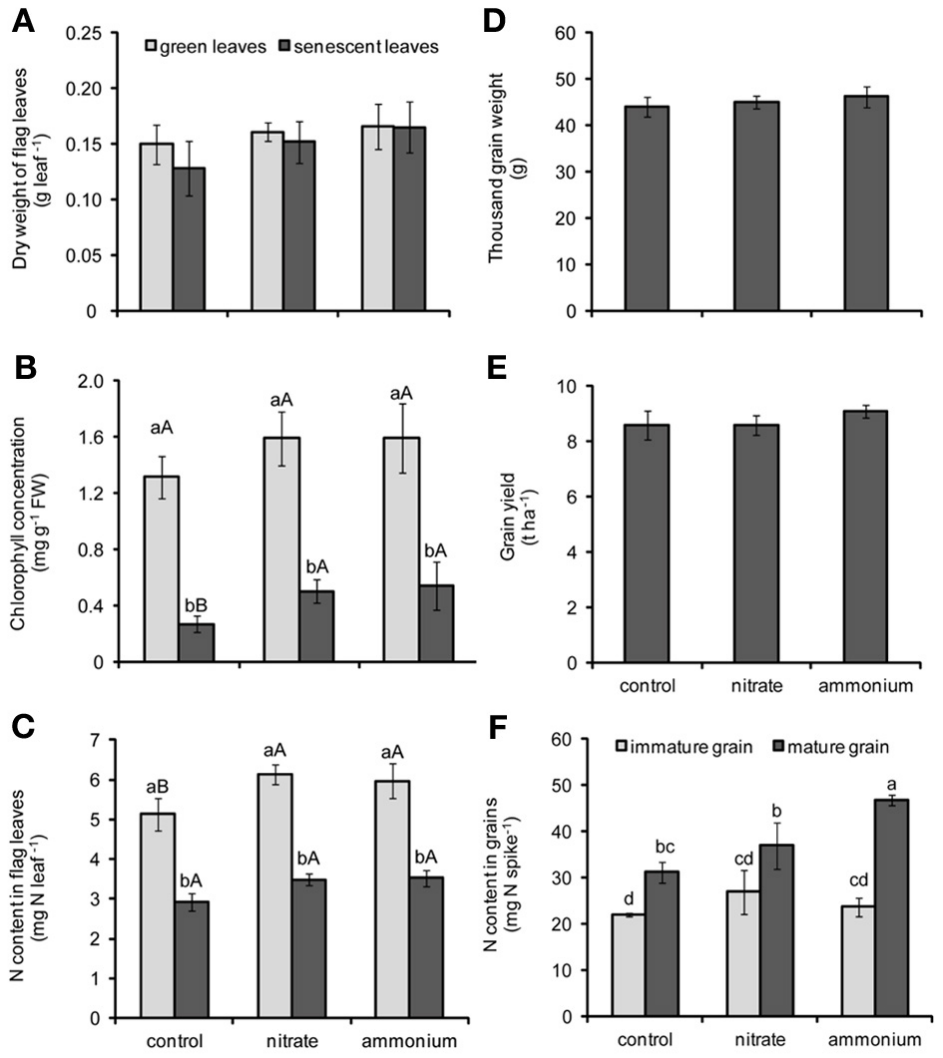
**Dry weight (A), chlorophyll concentrations (B), and N contents in flag leaves (C), thousand grain weight (D), grain yield (E), and N contents in grains (F) of winter wheat as affected by the supply of nitrate- or ammonium-based fertilizer (80 kg N ha^−1^) at anthesis (EC65).** Bars represent the means of 4 independent replicates ± SD, and 4 plants per replicate. Means followed by different letters indicate significant differences between treatments according to One-Way **(D**,**E)** or Two-Way ANOVA **(A,B,C**,**F)** followed by Tukey's test (*P* ≤ 0.05). In **(B)** and **(C)**, different lower-case letters indicate significant differences between harvest points within each N treatment, whereas different upper-case letters indicate significant differences among N forms within each harvest point (green or senescent leaves; immature or mature grains). The absence of letters indicates no significant difference for main factors or their interaction according to One-Way **(D**,**E)** or Two-Way **(A)** ANOVA.

To verify whether the chosen N fertilizer forms were effective in providing a nitrate- or ammonium-based fertilization, we extracted soil samples 5, 10, and 15 days after fertilizer application for the analysis of nitrate, ammonium, and urea. Ammonium nitrate application enhanced nitrate concentrations in the soil solution to >4 mM over all sampling days, while they remained at ~1 mM in unfertilized soil or soil supplemented with urea + nitrification inhibitor (Figure 1A). In general, ammonium concentrations were up to two orders of magnitude lower than those of nitrate (Figure 1B), indicating that ammonium nitrate application clearly provided nitrate as the dominant N form. The application of urea + nitrification inhibitor was effective in enhancing ammonium concentrations in the soil solution from 15 to 20 μM as in unfertilized soil samples to 25–30 μM, however, this level was also reached after ammonium nitrate application. By contrast, urea concentrations were elevated only 5 days after application of urea + nitrification inhibitor, providing proof for the limited stability of urea in the absence of an urease inhibitor (Figure 1C). These data suggested that the ammonium generated from the degradation of urea was rapidly and strongly adsorbed by the soil matrix. Such a high ammonium buffering capacity was not unexpected with regard to the high CEC of the soil (Table 1). As discussed below, the analysis of N and amino acids in the leaf samples were significantly increased after application of urea + nitrification inhibitor, in part even above the levels in ammonium nitrate-supplied plants, which is a typical response to ammonium-based fertilization (Figure 2C, Table 2). Thus, we concluded that the present methodological approach was valid to assign the ammonium nitrate application as a primarily nitrate-based fertilization, while the application of urea + nitrification inhibitor reflected more an ammonium-based fertilization.

### Influence of N fertilization on nutrient contents flag leaves

In order to assess whether a supply with the two N forms—ammonium and nitrate—at EC65 had a distinctive effect on plant development or leaf senescence of wheat plants, flag leaves were harvested at two time points after broadcast application of the respective N fertilizers. According to the results of Two-Way ANOVA, chlorophyll concentrations were significantly affected by both N fertilization and leaf developmental stage (Table 2). In all treatments, leaves harvested at the later stage (EC85) were chlorotic and exhibited significantly lower chlorophyll concentrations than those from the earlier harvest (EC75; Figure 2B), reflecting the progression of leaf senescence. Importantly, this effect was not related to changes in the biomass of flag leaves, since this parameter was not altered by the leaf developmental stage or N treatments (Figure 2A). Irrespective of the fertilized N form, N supply maintained higher N contents in green leaves and higher chlorophyll concentrations in senescing leaves (Figures 2B,C). Thus, both N forms appeared to delay leaf senescence to a similar extent.

In addition to total N, also the levels of amino acids were measured in flag leaves. As expected, the levels of all amino acids markedly decreased during flag leaf senescence (Table 3). The supply of both N forms increased the concentrations of glutamine and asparagine, whereas ammonium increased also the levels of aspartate in green leaves. However, the promoting effect of N fertilization on the amino acid pool declined at senescence. Even though the two N forms caused similar effects, ammonium-based fertilization led to a longer-lasting and more pronounced elevation of the amino acid pool which expressed particularly in a higher asparagine and aspartate accumulation in green leaves (Table 3). With regard to the higher amino acid accumulation usually seen under ammonium nutrition (Lang and Kaiser, 1994), this observation supported the view that the application of urea + nitrification inhibitor reflected a more ammonium-based N fertilization relative to the application of ammonium nitrate.

**Table 3:** **Effect of N forms on the concentration of amino acids in flag leaves of winter wheat (*Triticum aestivum* L. cv. Akteur)**.

**Treatments**	**Total free amino acids (μmol g^−1^ FW)**		**Glutamine (nmol g^−1^ FW)**		**Asparagine (nmol g^−1^ FW)**		**Glutamate (nmol g^−1^ FW)**		**Aspartate (nmol g^−1^ FW)**	
	**Green**	**Senescent**	**Green**	**Senescent**	**Green**	**Senescent**	**Green**	**Senescent**	**Green**	**Senescent**
Control	9.5 aB	2.6 bA	379 b	193 b	163 c	29 c	2132 a	747 b	940 aB	509 bA
Nitrate	15.2 aA	3.9 bA	1071 a	273 b	1450 b	74 c	2660 a	695 b	1734 aB	545 bA
Ammonium	16.1 aA	5.0 bA	1035 a	413 b	2995 a	45 c	4114 a	1113 b	2100 aA	853 bA

Ammonium- or nitrate-based N fertilizers were supplied at anthesis (80 kg N ha^−1^). Leaves were harvested at EC75 (green) or EC85 (senescent). Values are means of four replicates. For total free amino acids and aspartate, means in rows followed by different lower-case letters and means in the columns followed by different upper-case letters are significantly different from each other according to Two-Way ANOVA followed by Tukey's test (P ≤ 0.05). For the other amino acids, means followed by different letters are significantly different from each other according to Two-Way ANOVA followed by Tukey's test (P ≤ 0.05).

### Influence of N fertilization on yield traits

Both N treatments did neither significantly affect thousand grain weight nor grain yield (Figures 2D,E). Since several reports have indicated that the remobilization of micronutrients is associated with the remobilization of N (Kutman et al., 2010, 2011, 2012; Shi et al., 2010; Erenoglu et al., 2011), N contents in grains were assessed and found to be significantly increased during grain maturity (Figure 2F). In addition, Two-Way ANOVA revealed a significant effect of the interaction between leaf developmental stage and N forms on N content in grains (Table 2). The application of N fertilizer tended to slightly increase the N contents of immature grains. In mature grains, however, ammonium-based fertilization increased grain N contents by >50%, while there was no significant increase after nitrate fertilization (Figure 2F). Notably, these higher N contents in ammonium-fertilized grains did not coincide with a more pronounced decrease in the N content of flag leaves (Figure 2C).

### Influence of N fertilization on micronutrient accumulation in flag leaves and grains

**Figure 3:**
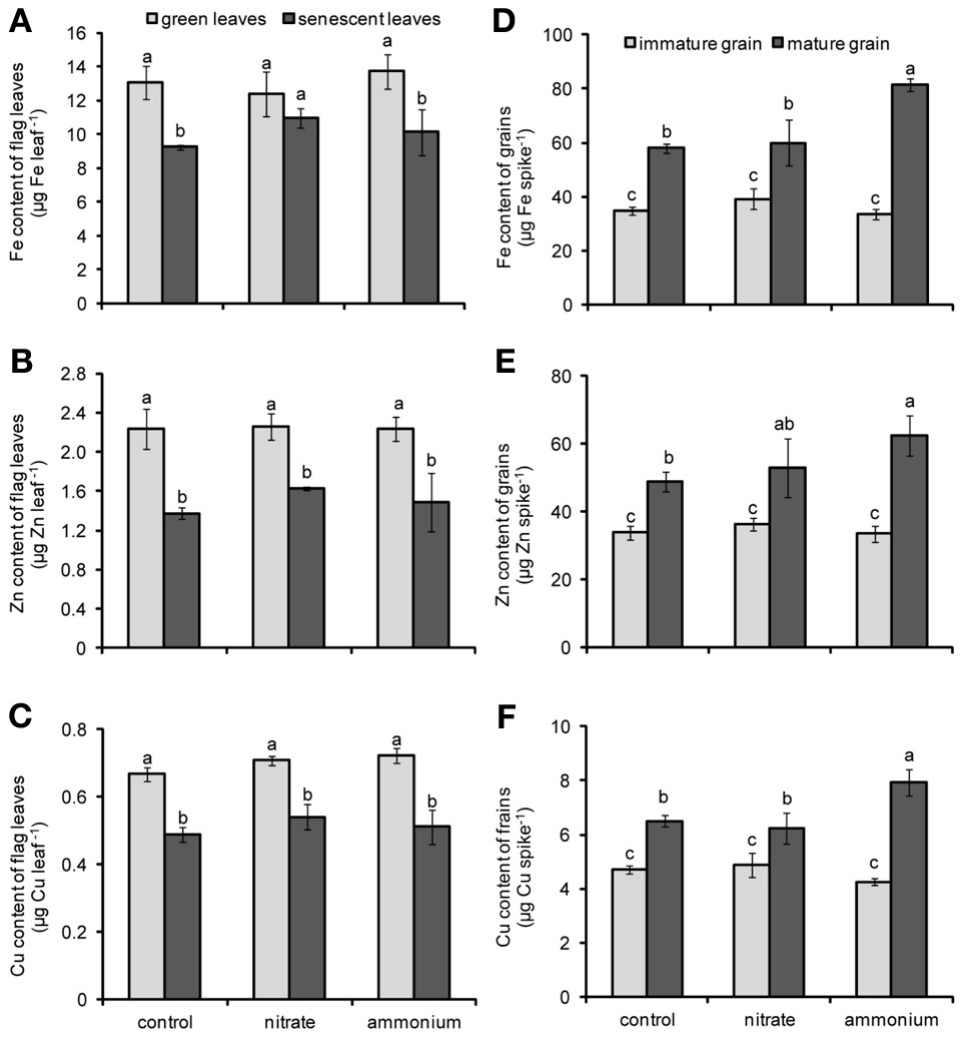
**Contents of Fe (A,D) Zn (B,E), and Cu (C,F) in flag leaves (A,B,C) or grains (D,E,F) of winter wheat as affected by nitrate- or ammonium-based fertilization (80 kg N ha^−1^) at anthesis (EC65).** Bars represent the means of 4 independent replicates ± SD, and 4 plants per replicate. Means followed by different letters indicate significant differences between treatments according to Two-Way ANOVA followed by Tukey's test (*P* ≤ 0.05).

According to the results of Two-Way ANOVA, the contents of Fe, Zn, and Cu in flag leaves were significantly affected only by the leaf developmental stage (Table 2). In fact, with progressing leaf senescence the contents of these micronutrients in flag leaves decreased significantly or at least in tendency as in case of Fe in nitrate-fertilized plants (Figures 3A,B,C). In general, this decrease indicated that these micronutrients were remobilized and exported out of the flag leaves during senescence. The supply of either N fertilizer to the plants did not significantly nor distinctively affect this process. The concentration of Fe, Zn and Cu were only slightly affected by the leaf developmental stage or the N fertilization (Figure A2). Regarding the contents of micronutrients in grains, Two-Way ANOVA revealed that not only the N fertilization and the leaf developmental stage, but also their interactions had significant effects on these traits (Table 2). The contents of Fe, Zn, and Cu increased as grains matured (Figures 3D,E,F). Importantly, the supply of a primarily ammonium-based fertilizer stimulated this increase only in mature but not in immature grains. Compared to non-fertilized and nitrate-supplied plants, the ammonium-based fertilization increased Fe contents in grains by almost 40% (Figure 3D) and those of Zn and Cu by 27 and 22%, respectively (Figures 3E,F). Thus, the increase in grain micronutrient contents after ammonium-based fertilization of wheat plants went along with a higher N content in the grains (Figure 2F) but not with a concomitant stimulation of micronutrient losses in flag leaves (Figures 3A–C).

### Influence of N fertilization on NA, DMA, and citrate levels

**Figure 4:**
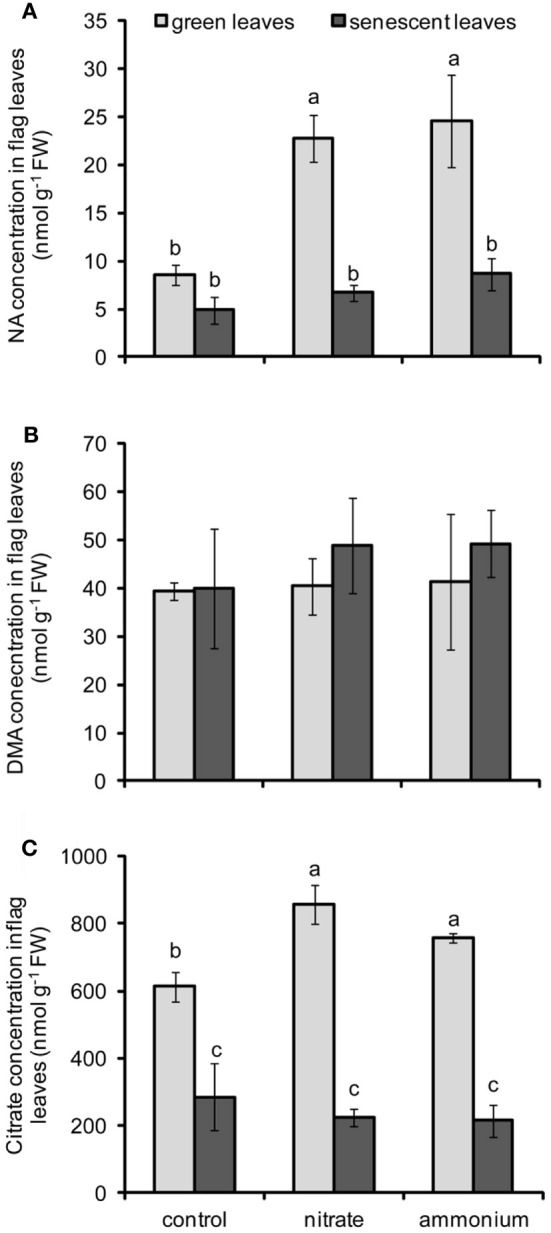
**Concentrations of NA (A), DMA (B), and citrate (C) in flag leaves of winter wheat as affected by nitrate- or ammonium-based fertilization (80 kg N ha^−1^) at anthesis (EC65).** Bars represent the means of 4 independent replicates ± SD, and 4 plants per replicate. Means followed by different letters indicate significant differences between treatments according to Two-Way ANOVA followed by Tukey's test (*P* ≤ 0.05). The absence of letters **(B)** indicates no significant difference for the main factors or their interaction according to Two-Way ANOVA.

The determination of micronutrient contents in flag leaves indicated that Fe, Zn, and Cu were remobilized from flag leaves during senescence (Figure 3). Since Fe, Zn, and Cu are assumed to be retranslocated via the phloem in complexed forms (Briat et al., 2007; Curie et al., 2009), changes in the concentrations of three major micronutrient ligands, namely NA, DMA, and citrate, were assessed in flag leaves. Whereas treatments had no significant effect on DMA levels, the main effects of N fertilization, leaf developmental stage and the effect of their interactions were significant for NA and citrate concentrations in leaves (Table 2). In case of NA, the supply of either N form strongly increased the concentration of NA in green flag leaves (Figure 4A) suggesting that the synthesis of this N-containing chelator was highly stimulated by N supply. In senescent flag leaves, NA concentrations were dramatically depleted in all treatments, even though NA levels remained slightly higher in ammonium-supplied plants (Figure 4A). In contrast, the levels of DMA only tended to be somewhat higher in senescing N-supplied leaves (Figure 4B). A striking difference to NA, however, was observed in the progression of DMA concentrations during senescence, when DMA levels did not decrease but stayed high. Similar to NA, also citrate levels decreased markedly during leaf senescence (Figure 4C) and N supply promoted citrate levels only in green but not any more in senescent leaves. However, there was no differential effect of the N form on citrate concentrations in flag leaves.

**Figure 5:**
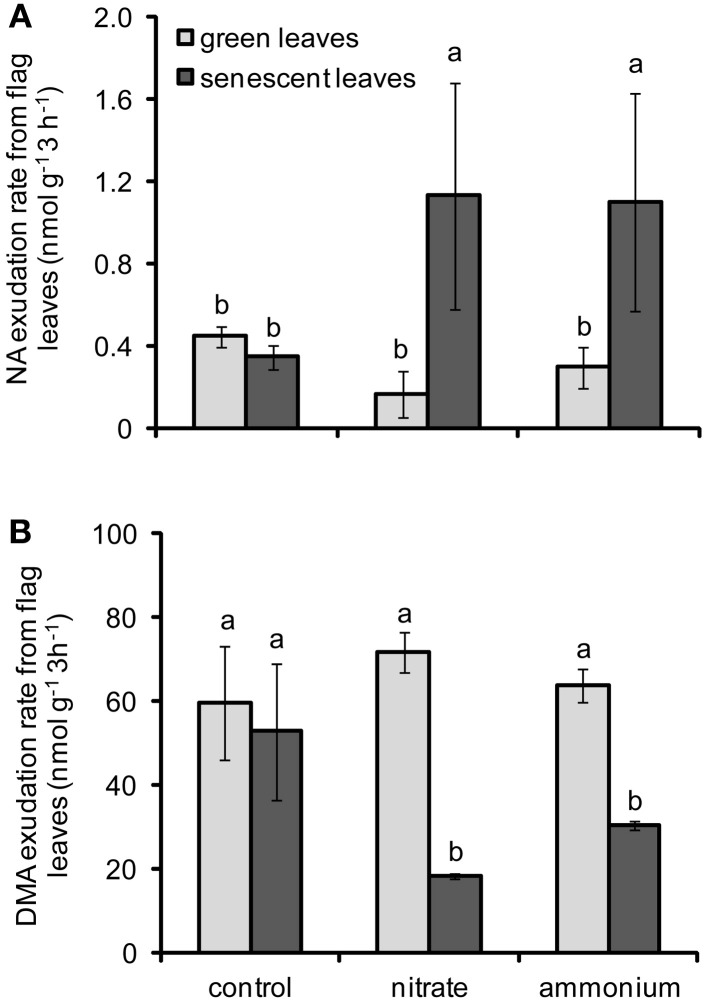
**Exudation rates of NA (A) and DMA (B) from flag leaves of winter wheat as affected by nitrate- or ammonium-based fertilization (80 kg N ha^−1^) at anthesis (EC65).** Bars represent the means of 4 independent replicates ± SD, and 4 plants per replicate. Means followed by different letters indicate significant differences between treatments according to Two-Way ANOVA followed by Tukey's test (*P* ≤ 0.05).

To gain an insight on the effects of N forms and leaf senescence on the retranslocation of micronutrient chelators, the levels of NA and DMA were also determined in exudates collected from flag leaves. Unfortunately, the amount of exudates collected was too small to determine citrate levels. The effect of the interaction between N fertilization and leaf developmental stage on NA and DMA exudation rates was significant (Table 2). In case of NA, N supply tended to decrease exudation rates from green leaves, whereas N supply increased the exudation of NA from senescent leaves (Figure 5A). Noteworthy, NA exudation rates from flag leaves of N-treated plants showed an opposite pattern as compared to the concentrations of NA detected in flag leaves (Figures 5A, 4A), further supporting that NA was exported out of these leaves during senescence. In contrast, N supply did not considerably affect the exudation of DMA from green flag leaves, but significantly reduced DMA export from senescent leaves of plants treated with either N form (Figure 5B). These results indicated that N supply stimulated the export of NA but not of DMA out of senescing flag leaves. Nevertheless, DMA export rates from flag leaves remained at least hundred-fold higher than those of NA at any developmental stage.

## Discussion

Earlier studies have indicated that an increasing N nutritional status of cereal plants can significantly improve Fe and Zn loading into grains. Thereby, high N supplies to wheat plants enhanced not only the acquisition and translocation of Fe and Zn to grains but also appeared to stimulate Zn retranslocation out of flag leaves and other plant organs (Kutman et al., 2010, 2011, 2012; Shi et al., 2010; Erenoglu et al., 2011). These studies indicated that N management is a promising route to improve micronutrient contents in cereal grains. The extent of remobilization, however, also depends on the nutritional status of the respective micronutrient. For instance, when plants were well-supplied with Fe, N supply decreased Fe retranslocation out of source leaves, probably because fertilized N created an additional sink for this metal in source leaves (Shi et al., 2012). Similarly, the amount of retranslocated Zn increased with N supply to a larger extent in Zn-deficient relative to Zn-sufficient wheat plants (Erenoglu et al., 2011). Thus, in order to obtain maximum gain in terms of micronutrient enrichment in grains, N fertilization has to be managed in a rational manner. Against this background, our study placed special emphasis on the influence of N fertilization and N fertilizer forms on metal and chelator pools in senescing source tissues, employing flag leaves as a relevant model organ for metal remobilization processes.

**Figure A1:**
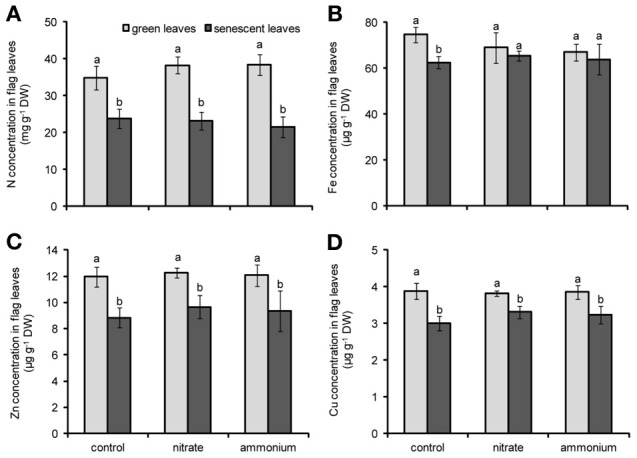
**Concentrations of N (A), Fe (B), Zn (C), and Cu (D) in flag leaves of winter wheat as affected by nitrate- or ammonium-based fertilization (80 kg N ha^−1^) at anthesis (EC65).** Bars represent the means of 4 independent replicates ± SD, and 4 plants per replicate. Means followed by different letters indicate significant differences between treatments according to Two-Way ANOVA followed by Tukey’s test (*P* ≤ 0.05).

**Figure A2:**
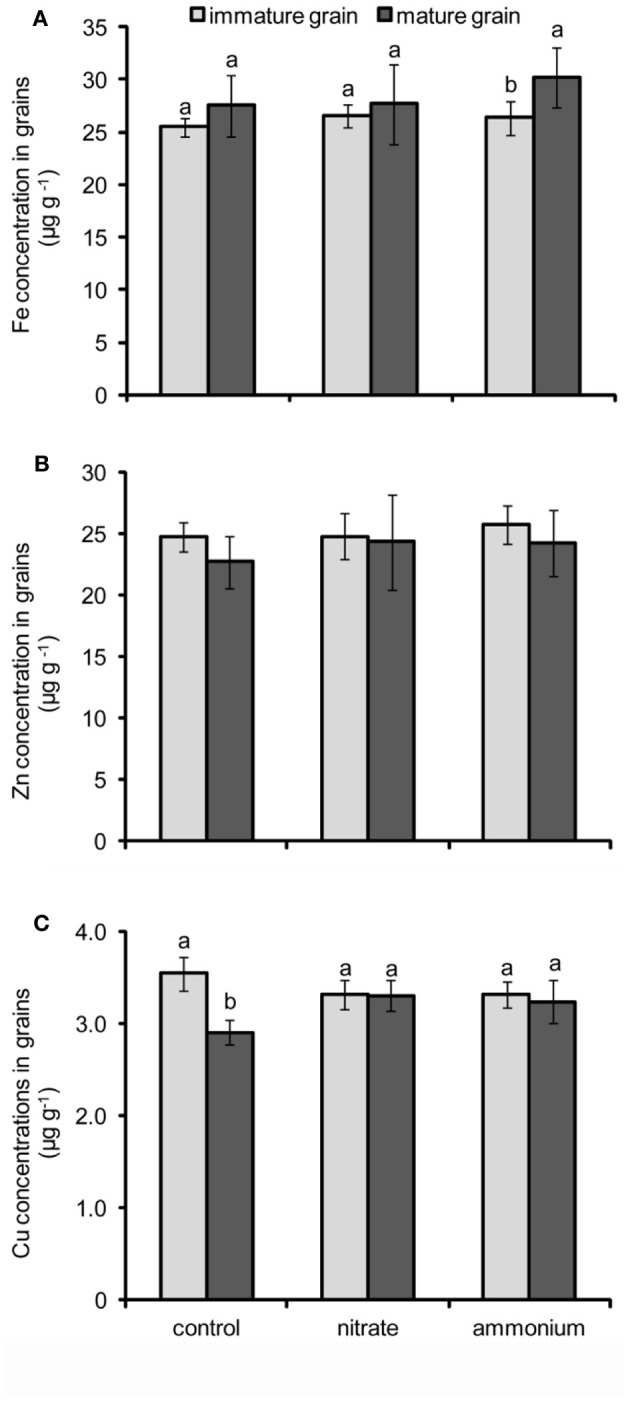
**Concentrations of Fe (A), Zn (B), and Cu (C) in grains of winter wheat as affected by nitrate- or ammonium-based fertilization (80 kg N ha^−1^) at anthesis (EC65).** Bars represent the means of 4 independent replicates ± SD, and 4 plants per replicate. Means followed by different letters indicate significant differences between treatments according to Two-Way ANOVA followed by Tukey’s test (*P* ≤ 0.05). The absence of letters **(B)** indicates no significant difference between treatments or their interactions according to Two-Way ANOVA.

As flag leaves started to senesce, it was observed that the contents of Fe, Zn, and Cu decreased significantly (Figures 3A,B,C), whereas they progressed to increase in developing grains (Figures 3D,E,F). In average, up to 20, 32, and 27% of flag leaf Fe, Zn, and Cu, respectively, were exported during leaf senescence (between stage EC75 and EC85). Previously, it has been reported that in sand-grown wheat up to 77% of shoot Fe was retranslocated to grains (Garnett and Graham, 2005). In contrast, in field-grown wheat Fe retranslocation during seed filling was negligible and not comparable to the values measured for Zn (Hocking, 1994). Such variable extents of metal retranslocation may relate to the nutritional status of the plants with the respective nutrients. In our case, soil pH was slightly alkaline and DTPA-extractable metal concentrations in the soil generally indicated low availabilities for Fe, Zn and Cu (Table 1). As a consequence Fe and Cu concentrations in green leaves were close to or, as in case of Zn, even slightly below the critical levels reported for wheat (Figure A1; Bergmann, 1992; Marschner, 2012). These data indicated that the nutritional status was favorable to promote metal retranslocation and supported the expectation that a beneficial and eventually differential effect of N fertilizer forms on metal retranslocation may be seen.

With regard to the possibility that different N forms may alter flag leaf metabolism or development and thereby micronutrient retranslocation, the most striking observation was that a primarily ammonium-based N fertilization after anthesis increased the accumulation of Fe, Zn, and Cu in grains (Figures 3D,E,F). The effect of ammonium on micronutrient accumulation was not related to a change in the progression of senescence, since ammonium-treated plants did not differ from nitrate-supplied plants with respect to chlorophyll or total N concentrations in the flag leaf at either the earlier or later harvest (Figures 2B,C). Nevertheless, slightly higher chlorophyll and leaf N concentrations after preferential nitrate- or ammonium-based fertilizer indicated that N fertilization was effective. Most of the N that ends up in wheat grains originates from the retranslocation of amino acids out of the vegetative organs, especially from flag leaves (Gregersen et al., 2008; Masclaux-Daubresse et al., 2008). A significant proportion of N can also be taken up from the soil after anthesis, if root activity has not yet ceased (Kichey et al., 2007). With regard to the low molecular-weight N pool that is usually dominated by amino acids, the supply of either inorganic N form significantly increased the concentrations of glutamine and asparagine in green flag leaves (Table 3). Since the extent of the depletion of Fe, Zn, and Cu in flag leaves of primarily ammonium- and nitrate-supplied plants was similar (Figures 3A,B,C), we consider it unlikely that the increased asparagine or slightly increased total amino acid levels in flag leaves and leaf exudates of ammonium-supplied plants were causally related to their elevated micronutrient contents in the grains. In another case, it has been shown that ammonium can promote Fe retranslocation from old to young leaves in maize (Zou et al., 2001), although this effect was more obvious when plants were grown under low Fe. Considering the growing evidence that Fe and Zn retranslocation out of flag leaves is tightly related to the retranslocation of N (Uauy et al., 2006; Shi et al., 2012), metal losses in senescent flag leaves were monitored. However, Fe, Zn, or Cu contents in flag leaves were not more decreased under preferential ammonium vs. nitrate nutrition (Figures 3A,B,C). Hence, the higher accumulation of these metal micronutrients in grains of ammonium-supplied plants (Figures 3D,E,F) was not caused by altered retranslocation processes. Moreover, control and nitrate-supplied plants accumulated similar amounts of these metals in the grain, suggesting that the total amount of added N fertilizer was not as relevant for metal accumulation in grains as the N fertilizer form. We therefore conclude that post-anthesis ammonium fertilization rather stimulated the *de novo* acquisition of micronutrient metals. A reason for this may be found in the rhizosphere acidification of ammonium-fertilized plants (Sarkar and Jones, 1982) or a longer lasting availability of ammonium-N, considering the high CEC of the soil used in this study (Table 1).

The root-to-shoot translocation in the xylem as well as the re-translocation of Fe and Zn in the phloem are facilitated by endogenous chelators such as NA and DMA, which are synthesized from methionine and are thus dependent on the N nutritional status. In agreement with this, we observed that a late application of N fertilizers to wheat plants significantly increased NA concentrations in flag leaves as well as NA exudation rates from senescing flag leaves (Figures 4A, 5A). In particular the latter suggested that in N-supplied plants more NA can be loaded into the phloem for long-distance transport. By contrast, DMA concentrations in the flag leaves were barely affected by N, and DMA export was even slightly repressed after N supply (Figures 4B, 5B). In rice plants, both of these chelators were found in the phloem sap: NA mainly as a Zn-NA complex and DMA predominantly complexed to Fe(III) (Nishiyama et al., 2012). Together with studies on transgenic, NAS-overexpressing plants (Lee et al., 2011), these reports emphasize the importance of NA and DMA for metal re-translocation. Unlike most other studies, our approach of collecting leaf exudates as an approximation for phloem sap additionally allowed comparing metal chelator availabilities and export from leaves at a quantitative level. In agreement with our previous observations in flag leaves of hydroponically-grown barley (Shi et al., 2012), we found much higher concentrations of DMA than of NA, not only in flag leaves but also in leaf exudates of field-grown wheat. Although this implies a far higher metal chelation potential by DMA, this may apply to Fe, Zn, and Cu in different ways with respect to their different complex stabilities (von Wirén et al., 1999). Moreover, the corresponding metal complexes are most likely also subject to differential phloem loading, since at least some of the YS1- and YSL-related transporters involved in long-distance metal-chelate transport may show differential preferences (Schaaf et al., 2004; Zheng et al., 2012). With regard to NA or DMA concentrations in leaves and in leaf exudates, none of the chelator pools investigated here responded differently to ammonium- or nitrate-based fertilization (Figures 4, 5). It is thus concluded that the N fertilizer form applied at anthesis had no particular impact on the mobilization and phloem loading of Fe, Zn, and Cu as well as their associated chelators in senescing flag leaves.

## Conclusions

Taken together, the present study found no evidence for N fertilization at anthesis increasing the export of Fe, Zn, and Cu from senescing flag leaves of wheat plants, although in particular NA pools responded positively to additional N supply. Similar to metal export, DMA concentrations were poorly affected by N fertilization. Nevertheless, DMA may be more relevant than NA for the mobilization and retranslocation of these metals in wheat, because far higher concentrations of DMA relative to NA were found in leaves and leaf exudates. Although the even higher concentrations of citrate may also contribute to metal retranslocation, it must be considered that metal-complex stabilities with citrate are several orders of magnitude lower than those of NA or DMA. While fertilized N forms did not affect metal retranslocation, the post-anthesis supply of ammonium-based fertilizer resulted in an elevated accumulation of these micronutrients in grains which was associated with a higher accumulation of N. This suggests that ammonium-based N fertilization was more effective than nitrate in stimulating the acquisition and translocation of micronutrient metals. To what extent N-dependent metal chelators contributed to these processes, requires further investigations.

### Conflict of interest statement

The authors declare that the research was conducted in the absence of any commercial or financial relationships that could be construed as a potential conflict of interest.

## Appendix

**Table A1:** **Two-Way analysis of variance (ANOVA) of the effects of N fertilization, leaf developmental stage and their interactions on response variables of field-grown winter wheat (*Triticum aestivum* cv. Akteur)**.

	**N**	**Leaf dev.**	**N fertil. × leaf**
	**fertilization**	**stage^a^**	**dev. stage**
N concentration of flag leaves	0.879	<0.001	0.573
Fe concentration of flag leaves	0.404	0.004	0.106
Zn concentration of flag leaves	0.466	<0.001	0.856
Cu concentration of flag leaves	0.419	<0.001	0.160
Fe concentration in grains	0.357	0.032	0.531
Zn concentration in grains	0.566	0.186	0.787
Cu concentration in grains	0.619	0.003	0.003

The F-value probabilities at 95% confidence are indicated.

a Refers to the developmental stage of either flag leaves (green or senescent) or grains (immature or mature).
